# Novel insight into histological and molecular astrocytoma, IDH‐mutant, Grade 4 by the updated WHO classification of central nervous system tumors

**DOI:** 10.1002/cam4.6476

**Published:** 2023-09-05

**Authors:** Wenlin Chen, Siying Guo, Yaning Wang, Yixin Shi, Xiaopeng Guo, Delin Liu, Yilin Li, Yuekun Wang, Hao Xing, Yu Xia, Junlin Li, Jiaming Wu, Tingyu Liang, Hai Wang, Qianshu Liu, Shanmu Jin, Tian Qu, Huanzhang Li, Tianrui Yang, Kun Zhang, Yu Wang, Wenbin Ma

**Affiliations:** ^1^ Department of Neurosurgery, Center for Malignant Brain Tumors, National Glioma MDT Alliance, Peking Union Medical College Hospital Chinese Academy of Medical Sciences and Peking Union Medical College Beijing China; ^2^ Eight‐year Medical Doctor Program Chinese Academy of Medical Sciences and Peking Union Medical College Beijing China; ^3^ China Anti‐Cancer Association Specialty Committee of Glioma Beijing China; ^4^ 4+4 Medical Doctor Program Chinese Academy of Medical Sciences and Peking Union Medical College Beijing China

**Keywords:** 2021 WHO classification of central nervous system tumors, astrocytoma, glioma, Grade 4, *IDH*‐mutant, molecular alteration, radiological characteristics

## Abstract

**Background:**

The latest fifth edition of the World Health Organization (WHO) classification of the central nervous system (CNS) tumors (WHO CNS 5 classification) released in 2021 defined astrocytoma, *IDH‐*mutant, Grade 4. However, the understanding of this subtype is still limited. We conducted this study to describe the features of astrocytoma, *IDH*‐mutant, Grade 4 and explored the similarities and differences between histological and molecular subtypes.

**Methods:**

Patients who underwent surgery from January 2011 to January 2022, classified as astrocytoma, *IDH*‐mutant, Grade 4 were included in this study. Clinical, radiological, histopathological, molecular pathological, and survival data were collected for analysis.

**Results:**

Altogether 33 patients with astrocytoma, *IDH*‐mutant, Grade 4 were selected, including 20 with histological and 13 with molecular WHO Grade 4 astrocytoma. Tumor enhancement, intratumoral‐necrosis like presentation, larger peritumoral edema, and more explicit tumor margins were frequently observed in histological WHO Grade 4 astrocytoma. Additionally, molecular WHO Grade 4 astrocytoma showed a tendency for relatively longer overall survival, while a statistical significance was not reached (47 vs. 25 months, *p* = 0.22). *TP53*, *CDK6*, and *PIK3CA* alteration was commonly observed, while *PIK3R1* (*p = 0.033*), *Notch1* (*p = 0.027*), *and Mycn (p = 0.027)* alterations may affect the overall survival of molecular WHO Grade 4 astrocytomas.

**Conclusions:**

Our study scrutinized *IDH*‐mutant, Grade 4 astrocytoma. Therefore, further classification should be considered as the prognosis varied between histological and molecular WHO Grade 4 astrocytomas. Notably, therapies aiming at *PIK3R1*, *Notch 1*, and *Mycn* may be beneficial.

## 1 INTRODUCTION

Glioma is the most common primary malignant tumor of the central nervous system (CNS), with an average annual incidence of 4.50 per 100,000.^1^ Current clinical treatment options for glioma include surgery, radiotherapy, chemotherapy, immunotherapy, targeted therapy, and tumor‐treating fields.^2^, ^3^ However, the prognosis remains unfavorable.^1^ Considering the poor prognosis status and the vacancy of widely recognized therapeutic targets, a more accurate classification standard or guideline is significantly needed for individualized treatment and prognosis prediction. The World Health Organization (WHO) initially integrated the classification and grading of CNS tumors in 1979.^4^ After several revisions and updates,^5^ the fourth edition of the WHO classification of CNS tumors (WHO CNS 4 classification) mainly focused on the histological characteristics of tumors.^6^, ^7^ Following the publication of the WHO CNS 4 classification guideline in 2016, researchers have identified several specific molecular markers including IDH1/2, EGFR, TERT, and MGMT that are relevant for survival and response to treatment in patients with CNS tumors.^8^, ^9^, ^10^, ^11^ However, the independent prognostic value of the molecular markers in all age groups remains to be determined.^12^ Based on these findings, the latest fifth edition of the WHO classification of CNS tumors (WHO CNS 5 classification) released in 2021 focused on advancing the role of molecular diagnosis in CNS tumor classification. Notably, it combines histopathology and molecular pathology for comprehensive diagnosis to further predict prognosis and conduct comprehensive treatment decisions.^13^

In the WHO CNS 5 classification criteria, the diagnosis of *IDH*‐mutant glioblastoma no longer exists. This type of tumor, previously known as “secondary glioblastoma”, is now defined as astrocytoma, *IDH*‐mutant, Grade 4. In addition to histological Grade 4 *IDH*‐mutant astrocytoma, astrocytoma with *IDH*‐mutations of WHO Grades 2, 3 and homozygous *CDKN2A* and/or *CDKN2B* deletions are also defined as astrocytoma, *IDH*‐mutant, Grade 4, despite their relatively lower histopathology grades.^8^, ^13^, ^14^, ^15^ *CDKN2A* encodes p14ARF and INK4a and plays a role in regulating tumor suppression.^16^, ^17^ While p14ARF inactivates *MDM2*, an E3 ubiquitin ligase of p53, and causes p53‐mediated cell‐cycle arrest or apoptosis, INK4a promotes pRB‐mediated cell‐cycle arrest.^18^, ^19^, ^20^, ^21^ Additionally, *CDKN2B* encodes INK4b, which inactivates *CDK4/CDK6* and controls cell cycle progression.^22^, ^23^ It is suspected that homozygous deletion of *CDKN2A/B*, which breaks the “brake” of tumor progression brake, nullifies the tumor‐suppressive environment created by *IDH*‐mutant astrocytomas.^24^, ^25^ However, on a biomolecular level, detailed interactive mechanisms between *CDKN2A/B* and *IDH1/2* remain unknown. Meanwhile, other molecular biomarkers indicating worse outcome in IDH‐mutant astrocytoma, including *NOTCH1* mutations and incomplete resections in oligocytoma and *PIK3R1* mutations, *RB1* mutations and *CDK4* mutations and amplifications in astrocytoma.^26^, ^27^ Although the molecular alteration characteristics of astrocytoma, *IDH*‐mutant, Grade 4 in the new classification have been partially explored, studies focused more on the differences between astrocytoma, IDH‐mutant, Grade 4 and glioblastoma.^28^, ^29^, ^30^, ^31^ There are few clinical studies that specifically focus on this subtype, and our understanding remains limited.^31^, ^32^

Increasing attention to the novel species of astrocytoma, *IDH*‐mutant, Grade 4 from the WHO CNS 5 classification has led to extensive research and exploration. In this study, we describe the clinical, radiological, and molecular pathological features of astrocytoma, *IDH*‐mutant, Grade 4 based on the WHO CNS 5 classification. Furthermore, we explored the similarities and differences between the two subtypes of histological WHO Grade 4 astrocytoma and molecular WHO Grade 4 astrocytoma and searched for predictive molecular markers to benefit patient prognosis. To do so, we analyzed our institution's comprehensive data on glioma patients. Notably, we aimed to provide a database for deepening the academic understanding of astrocytoma, *IDH*‐mutant, Grade 4, and clinical treatment options.

## 2 METHOD

### 2.1 Study population

Patients who underwent surgery at the Department of Neurosurgery at Peking Union Medical College Hospital from January 2011 to January 2022,^33^ classified as astrocytoma, *IDH*‐mutant, Grade 4 according to WHO CNS 5 classification criteria, were screened for this study. Finally, patients with complete clinical and pathological data were included in our analysis. Written informed consent was obtained from all patients included for the use of clinical samples. The study was approved by the Institutional Ethics Review Board of PUMCH (S‐424) and conformed to the requirements of the Declaration of Helsinki.

### 2.2 Clinical and radiological data collection

Clinical and radiological information was collected retrospectively from patients' medical records and examinations. Patient clinical information utilized in our analyses included gender, age at diagnosis, body mass index (BMI), preoperative Karnofsky performance status (KPS) score, clinical symptoms, the extent of operation resection (EOR), and postoperative comprehensive treatment regimen. In addition, we collected patient survival data and adverse events through outpatient and telephone follow‐ups, with overall survival (OS) defined as the time from the surgery date to the patient's death or last‐recorded follow‐up (treated as censored values). The median OS was defined as the overall survival achieved by 50% of patients.

Radiological features of involved patients were collected from preoperative magnetic resonance imaging (MRI) to analyze the number of tumors, tumor location, involvement of functional areas, maximum tumor diameter, maximum edema diameter, intratumoral‐necrosis like presentation, intratumoral hemorrhage, cystic lesions, calcification, tumor margin clarity, and tumor performance in T1 weighted image (T1WI), T2WI, and T1‐based contrast‐enhanced images. In contrast‐enhanced MRI, the boundary between the tumor parenchyma and the edema area near the tumor or adjacent brain tissue is clearly defined as a clear tumor boundary. If it was not discernible by the naked eye, the tumor boundary was defined as blurred.^34^

### 2.3 Tumor pathology data collection

Histopathological and molecular pathological data were collected. Histopathological data were obtained from the Department of Pathology of Peking Union Medical College Hospital report, mainly including the Ki‐67 index and tumor histological grade. For molecular pathology, formalin‐fixed paraffin‐embedding (FFPE) tumor samples were collected from enrolled patients. DNA extraction was conducted with QIAGEN 56404 Kit, and nucleotide concentration and purity were determined by Qubit 4.0 Fluorometer (Thermo Fisher Scientific) using the dsDNA HS Assay Kit and Nanodrop 2000 spectrophotometer (Thermo Fisher Scientific) respectively. We screened 60 molecular markers, including *EGFR*, *TERT*, *CDKN2A/B*, *MYB*, and *MYBL1*, by summarizing the recently published studies on the mechanism of glioma development and prognostic factors. DNA extraction was conducted with QIAGEN 56404 Kit, and nucleotide concentration and purity were determined by Qubit 4.0 Fluorometer (Thermo Fisher Scientific) using the dsDNA HS Assay Kit and Nanodrop 2000 spectrophotometer (Thermo Fisher Scientific) respectively. We analyzed the molecular alterations of each enrolled patient using next‐generation sequencing (NGS), and used polymerase chain reaction (PCR)‐based assays and fluorescence in situ hybridization methods (FISH) for verification. CNVkit was applied to define the copy number variation from DNA sequencing results. The objective criteria for copy number loss were the ratio of copy number < = 1.5 while the number of bin > = 0.3, and the objective criteria for copy number gain were the ratio of copy number > = 2.5 while the number of bin > = 0.3.^35^ The complete list of molecular markers is shown in Table S1.

### 2.4 Identification of enrolled patients and group analysis according to WHO CNS5

According to the WHO CNS 5 classification criteria, astrocytoma, *IDH*‐mutant, Grade 4 includes histological WHO Grade 4 astrocytoma with necrosis or microvascular proliferation (histological astrocytoma as defined in this study) and WHO Grades 2, 3 astrocytomas included homozygous *CDKN2A* and/or *CDKN2B* deletions (molecular astrocytoma as defined in this study, further examinations were conducted to ensure the homozygous deletion status for *CDKN2A/B*). We further explored the differences and similarities between the two subtypes regarding clinical information, radiological features, prognostic status, and molecular alterations.

### 2.5 Statistical analysis

Normally distributed measures were expressed as means ± standard deviations (SDs), and student's *t*‐test determined differences between groups. Non‐normally distributed measures were expressed as medians (first quartile, third quartile) (M [Q1, Q3]) and analyzed between groups by the Kruskal–Wallis *H* test. Additionally, comparisons of categorical variables were performed using the chi‐squared test. Molecular alterations in histological and molecular WHO Grade 4 astrocytoma were presented using oncoplots. Survival analysis was performed using the Kaplan–Meier method and Log‐Rank test, and Kaplan–Meier curves presented the results. *p* < 0.05 was considered to be statistically significant. All statistical analyses were performed with SPSS (version 26.0, IBM) statistical software, and graphs were created using R Studio (PBC & Certified B Corp.®) and GraphPad Prism (9, GraphPad Software) software.

## 3 RESULTS

### 3.1 Baseline information of patients with astrocytoma, *IDH* ‐mutant, Grade 4

A total of 33 patients (27 males and 6 females) with astrocytoma, *IDH*‐mutant, Grade 4 were involved in this study, including 20 with histological and 13 with molecular astrocytoma. The mean age was 44 years, and the mean preoperative KPS was 83. In terms of clinical symptoms, 19 (57.6%) emerged with neurological deficits, and 15 (45.5%) exhibited seizures. For EOR, 57.6% (19/33) of patients received gross total resection, 27.3% (10/33) underwent subtotal resection, and 15.1% (5/33) experienced biopsy. For combination therapy, 20 patients received chemotherapy, 19 completed radiotherapy, and 6 underwent targeted treatment. Notably, statistical significances were observed in gender, histological grading, and Ki‐67 levels, while the difference in gender may due to the limitation of sample size. The specific information is shown in Table 1.

**TABLE 1:** Baseline characters of 33 patients with histological or molecular astrocytoma, *IDH*‐mutant, Grade 4.

	All astrocytoma, *IDH*‐mutant, Grade 4	Histological astrocytoma, *IDH*‐mutant, Grade 4	Molecular astrocytoma, *IDH*‐mutant, Grade 4	*p* value
Gender				**0.010**
Male	27, 81.8%	14, 70.0%	13, 100.0%	
Female	6, 18.2%	6, 30.0%	0	
Mean Age, year	44.12 ± 12.78	47.10 ± 14.23	39.54 ± 8.80	0.097
Age, year				0.211
<18	0	0	0	
18–44	19, 57.6%	10, 50.0%	9, 69.2%	
45–64	10, 30.3%	6, 30.0%	4, 30.8%	
≥65	4, 12.1%	4, 20.0%	0	
Mean BMI, kg/m^2^	23.82 ± 2.48	24.21 ± 2.45	23.22 ± 2.51	0.267
BMI, kg/m^2^				0.619
<18	0	0	0	
18–24	17, 51.5%	11, 55.0%	6, 46.2%	
≥24	16, 48.5%	9, 45.0%	7, 53.8%	
Preoperative KPS	83.03 ± 17.94	84.50 ± 14.31	80.76 ± 22.90	0.568
Clinical symptoms				
Neurologic impairment	19, 57.6%	14, 70.0%	5, 38.5%	0.077
Seizure	15, 45.5%	9, 45.0%	6, 46.2%	0.950
Histological grade classification				**<0.001**
WHO Grade 2	5, 15.1%	0	5, 38.5%	
WHO Grade 3	8, 24.3%	0	8, 61.5%	
WHO Grade 4	20, 60.6%	20, 100.0%	0	
Ki‐67	30.00% (10.00%, 60.00%)	30.00% (20.00%, 70.00%)	8.00% (3.00%, 35.00%)	**0.024**
Extent of surgical resection				0.125
Gross total resection	19, 57.6%	14, 70.0%	5, 38.5%	
Subtotal resection	9, 27.3%	3, 15.0%	6, 46.2%	
Biopsy	5, 15.1%	3, 15.0%	2, 15.3%	
Postoperation therapy				
Chemotherapy	20, 60.6%	12, 60.0%	8, 61.5%	0.932
Radiotherapy	19, 57.6%	11, 55.0%	8, 61.5%	0.721
Targeted Therapy	6, 18.2%	5, 25.0%	1, 7.7%	0.178

*Note*: Significant values are presented in bold.

### 3.2 Radiological features of patients with astrocytoma, *IDH* ‐mutant, Grade 4

**FIGURE 1:**
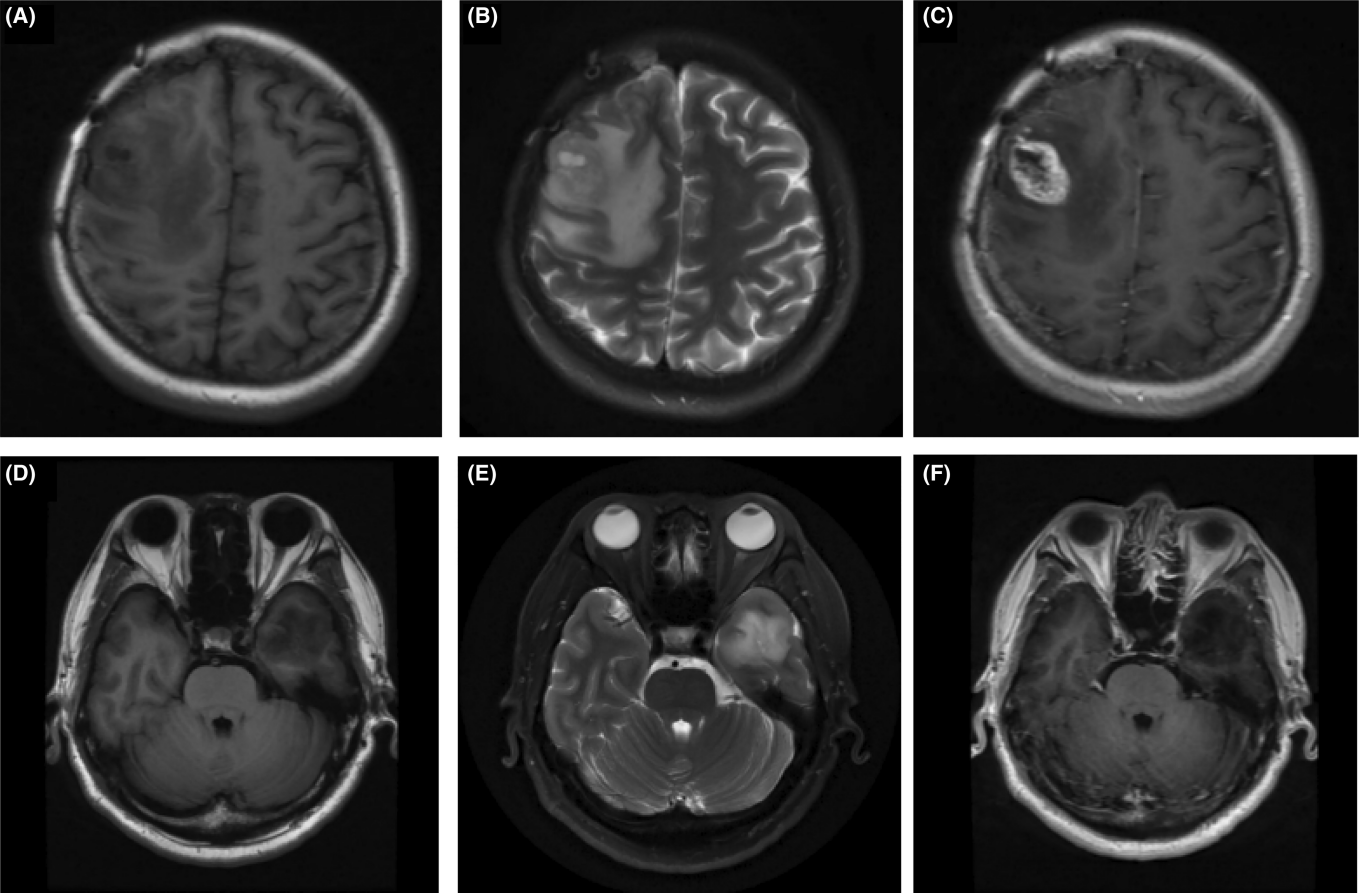
(A–C) A 31‐year‐old female with astrocytoma, *IDH*‐mutation, Grade 4 without *CDKN2A/B* deletion, with rim enhancement; (D–F) A 46‐year‐old male with astrocytoma, *IDH*‐mutation, Grade 4 with *CDKN2A/B* deletion, without enhancement.

The essential diagnostic basis for intracranial tumors in clinical diagnosis is neurological imaging. We summarized and compared the radiological characteristics of patients in the histological and molecular astrocytoma groups. Due to incomplete radiological data for some patients, 14 patients with histological WHO 4 grade astrocytoma and 12 patients with molecular WHO 4 grade astrocytoma were ultimately included, and typical radiological images were shown (Figure 1).

Regarding tumor distribution, solitary tumors occurred more frequently than multiple tumors in both groups, and there were no significant differences in tumor side, specific lobar localization, and functional area involvement. MRI in both groups demonstrated low or mixed density on T1W1 and high density on T2W1. At the same time, in the contrast‐enhanced image, enhancement was present in 92.9% (13/14) of patients in the histological astrocytoma group and only 33.3% (4/12) of patients in the molecular astrocytoma group. No differences were observed in the histological and molecular WHO Grade 4 astrocytomas in the rate of intratumoral hemorrhage, cystic degeneration, and calcification. In contrast, the histological astrocytoma group showed larger peritumoral edema, higher incidence of intratumoral‐necrosis like presentation, and more explicit tumor margin. However, statistical significance was not reached (*p* values were 0.186, 0.086, and 0.052, respectively). Table 2 summarizes the radiological features of WHO Grade 4 astrocytomas.

**TABLE 2:** Radiological characters of 26 patients with histological or molecular astrocytoma, *IDH*‐mutant, Grade 4. Seven patients were excluded from this analysis due to a lack of radiological information.

	All astrocytoma, *IDH*‐mutant, Grade 4	Histological astrocytoma, *IDH*‐mutant, Grade 4	Molecular astrocytoma, *IDH*‐mutant, Grade 4	*p* value
Number of tumors				0.652
Solitary	23, 88.5%	12, 85.7%	11, 91.7%	
Multiple	3, 11.5%	2, 14.3%	1, 8.3%	
Side of the tumor				0.908
Left hemisphere	11, 42.3%	5, 35.7%	6, 50.0%	
Right hemisphere	10, 38.5%	7, 50.0%	3, 25.0%	
Bilateral	5, 19.2%	2, 14.3%	3, 25.0%	
Tumor location				0.664
Frontal lobe	10, 38.5%	6, 42.9%	4, 33.3%	
Parietal lobe	0	0	0	
Temporal lobe	2, 7.7%	1, 7.1%	1, 8.3%	
Occipital lobe	0	0	0	
Multiple lobes involved	14, 53.8%	7, 50.0%	7, 58.4%	
Tumor in functional region				0.347
Motor functional area	4, 15.4%	3, 21.4%	1, 8.3%	
Sensory functional area	2, 7.7%	1, 7.1%	1, 8.3%	
Language functional area	1, 3.8%	1, 7.1%	0	
None	19, 73.1%	9, 64.3%	10, 83.3%	
Maximum diameter of the tumor, cm	4.67 ± 1.39	4.23 ± 1.23	5.24 ± 1.44	0.083
T1 weighted image				0.473
Hypointensity	17, 65.5%	10, 71.4%	7, 58.4%	
Heterogeneous intensity	7, 26.9%	4, 28.6%	3, 25.0%	
Hyperintensity	1, 3.8%	0	1, 8.3%	
Unknown	1, 3.8%	0	1, 8.3%	
T2 weighted image				0.482
Hypointensity	0	0	0	
Heterogeneous intensity	11, 42.3%	5, 35.7%	6, 50.0%	
Hyperintensity	15, 57.7%	9, 64.3%	6, 50.0%	
Contrast‐enhanced MRI				**0.001**
Not enhanced	8, 30.8%	1, 7.1%	7, 58.4%	
Enhanced	17, 65.4%	13, 92.9%	4, 33.3%	
Unknown	1, 3.8%	0	1, 8.3%	
Diameter of edema expansion, cm	3.15 ± 5.93	4.74 ± 7.95	1.41 ± 1.28	0.186
Intratumoral‐necrosis like presentation*				0.086
Yes	14, 53.8%	10, 71.4%	4, 33.3%	
No	11, 42.3%	4, 28.6%	7, 58.4%	
Unknown	1, 3.8%	0	1, 8.3%	
Intratumoral bleeding				0.759
Yes	5, 19.2%	3, 21.4%	2, 16.7%	
No	21, 80.8%	11, 78.6%	10, 83.3%	
Cystic degeneration				0.793
Yes	8, 30.8%	4, 28.6%	4, 33.3%	
No	18, 69.2%	10, 71.4%	8, 66.7%	
Calcification				0.271
Yes	1, 3.8%	0	1, 8.3%	
No	25, 96.2%	14, 100.0%	11, 91.7%	
Clarity of tumor margin				0.052
Yes	14, 53.8%	10, 71.4%	4, 33.3%	
No	12, 46.2%	4, 28.6%	8, 66.7%	

*Note*: Significant values are presented in bold.

* Intratumoral‐necrosis like presentation refers to the MRI images presented with hypointensity or heterogeneous intensity on T1WI and hyperintensity or heterogeneous intensity on T2WI.

### 3.3 Survival of patients with astrocytoma, *IDH* ‐mutant, Grade 4

**FIGURE 2:**
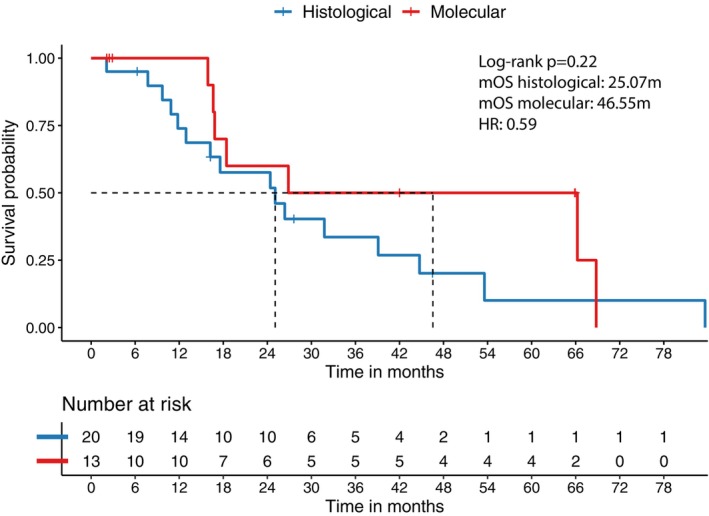
Kaplan–Meier curve showing distinct overall survival between patients with astrocytoma, *IDH*‐mutation, WHO 4 without *CDKN2A/B* deletion and patients with astrocytoma, *IDH*‐mutation with *CDKN2A/B* deletion (mOS: 25.1 vs. 46.6 m, *p* = 0.22).

In both subgroups, we further explored the survival of patients with astrocytoma, *IDH*‐mutant, Grade 4. The mOS of histological astrocytoma was 25 months. In comparison, the mOS of molecular astrocytoma was 47 months, suggesting that molecular astrocytoma was more likely to have a better prognosis and more prolonged survival than the histological subgroup. However, no statistical significance was observed in the analysis due to the limited sample size (*p* = 0.22). These results and specific survival information are presented in Figure 2.

### 3.4 Molecular features in patients with astrocytoma, *IDH* ‐mutant, Grade 4

**FIGURE 3:**
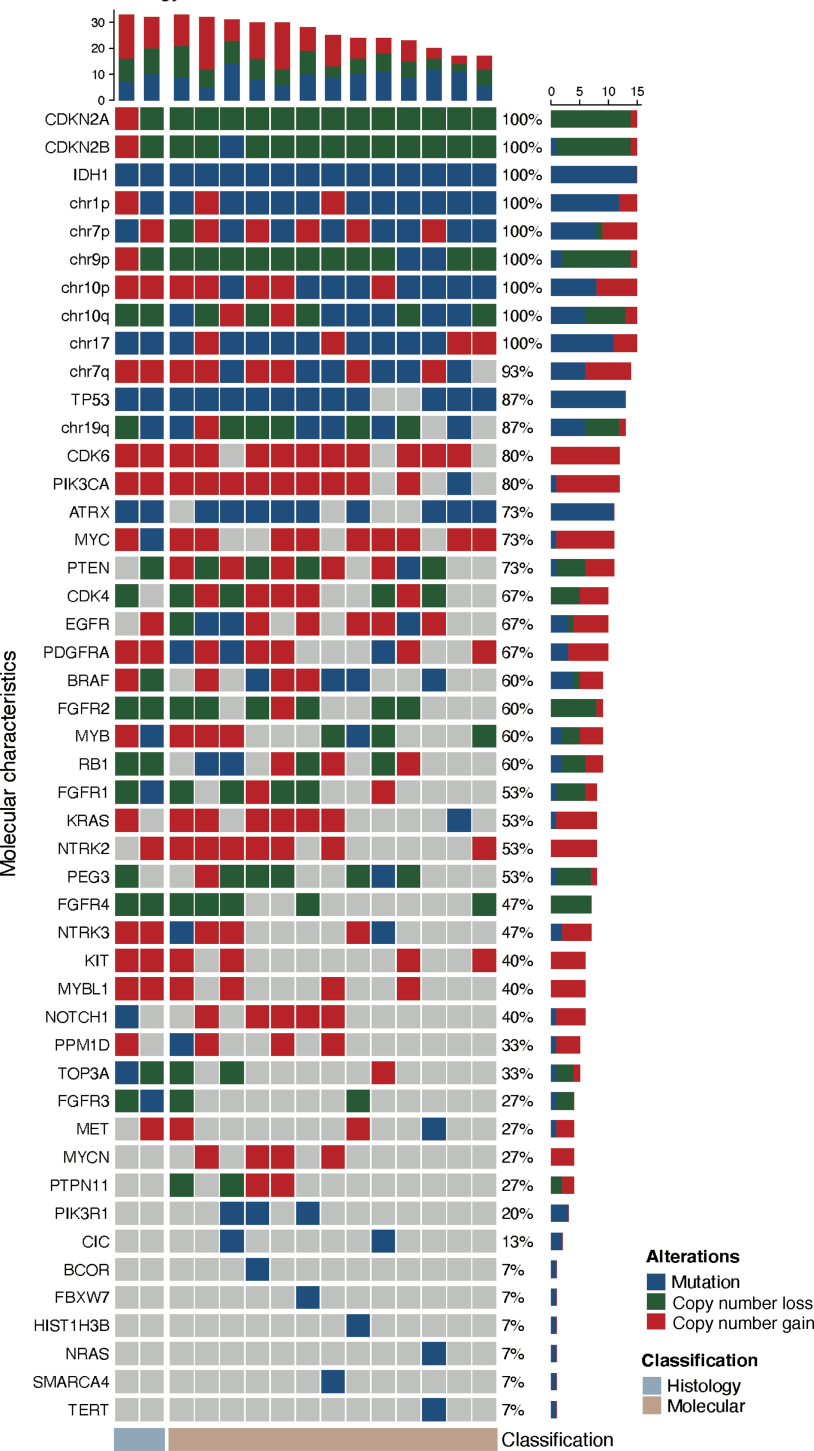
Oncoplot of the molecular alterations of the 15 patients with histological or molecular astrocytoma, *IDH*‐mutant, Grade 4 based on the WHO 2021 classification of CNS tumors. The copy number variation was defined with CNVkit, and copy number gain was defined as the ratio of copy number ≥ 2.5 while the number of bin ≥ 0.3, and copy number loss was defined as the ratio of copy number ≤ 1.5 while the number of bin ≥ 0.3. Each column represents an individual patient, and the cube's color indicates the alteration status of each molecular characteristic. Blue: Mutation; Green: Copy number loss; Red: Copy number gain; Gray: Histological astrocytoma, *IDH*‐mutant, Grade 4; Brown: Molecular astrocytoma, *IDH*‐mutant, Grade 4.

Different patterns of molecular marker alterations exist in different gliomas, including mutations, copy number gains, or copy number losses of chromosomes, and genes. Notably, we analyzed and demonstrated the molecular alterations of tumors in two subgroups. As some patients did not undergo molecular pathological examination due to incomplete specimens, we ultimately included two patients with histological WHO Grade 4 astrocytoma and 13 with molecular WHO Grade 4 astrocytoma. Figure 3 shows the changes in molecular markers in both groups of patients. In addition to alterations in *IDH1* and *CDKN2A/B*, common genetic alterations in patients with WHO Grade 4 astrocytoma included *TP53*, *CDK6*, and *PIK3CA*.

### 3.5 Implications of molecular alterations with survival in patients with astrocytoma, *IDH* ‐mutant, Grade 4

**FIGURE 4:**
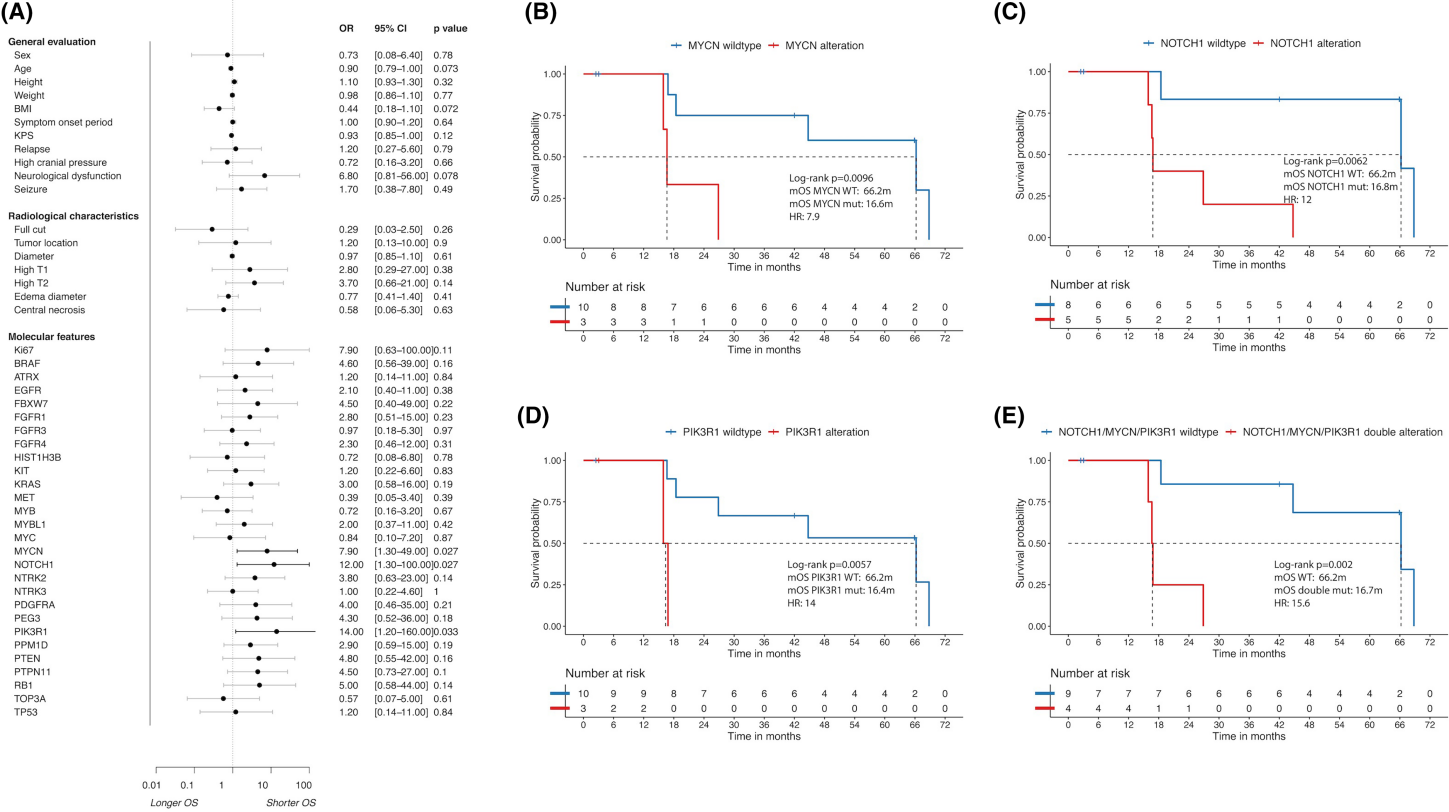
*PIK3R1/Notch1/Mycn* were identified as important prognostic factors for molecular WHO Grade 4 astrocytoma. Thirteen patients were included. (A) Forest plot of univariate Cox analysis. The plot also shows the hazard ratio (HR), 95% confidence interval (CI), and the *p* value of each variable. (B–E) Kaplan–Meier curves showing distinct median OS in populations with or without *PIK3R1/Notch1/Mycn* alteration.

Currently, molecular markers for clinical classification, prognosis, and therapeutic response prediction of astrocytoma include *MGMT* promoter methylation and homozygous *CDKN2A* and/or *CDKN2B* deletions.^8^, ^36^, ^37^ This study focused on the molecular pathology of astrocytoma, *IDH*‐mutant, Grade 4. We also explored other clinical and molecular marker alterations associated with patient prognosis, providing clues for prognostic prediction and clinical decision‐making. The results of univariate regression analysis suggested that alterations in PIK3R1 (*p* = 0.033), Notch1 (*p* = 0.027), and Mycn (*p* = 0.027) may be associated with shorter OS in molecular WHO Grade 4 astrocytoma. Likewise, patients with double genetic alterations also showed worse OS in the subgroup analysis (Figure 4, *p* = 0.002). We further analyzed the effect of other clinical and molecular marker alterations on survival in patients with histological and molecular WHO Grade 4 astrocytoma. However, no correlation was seen between other clinical and molecular marker alterations and the survival of patients (Figure [Link], [Link]).

## 4 DISCUSSION

In light of the WHO CNS 5 classification criteria, new insights and more precise recognitions have been made regarding gliomas. This study focused on astrocytoma, *IDH*‐mutant, Grade 4, whose definition was considerably changed based on the latest WHO classification. Thirty‐three patients were included, and differential clinical features were observed in histological and molecular subgroups. Detailed surgical plans including parameters concerning the localization of tumors, the presence of deep lesions, and the presence of seizures at onset, should facilitate a thorough analysis of the glioma.^38^ Additionally, significantly different sex distribution was observed due to the limited cohort size. For the radiological manifestations, astrocytoma *IDH*‐mutant, Grade 4 typically shows T1 hypodensity, T2 hyperintensity, and rim enhancement around central necrosis, while glioblastomas are usually irregularly shaped, with ring‐enhancement, central necrosis, and surrounding oedema. In terms of subgroup analysis, tumor enhancement in the T1 enhancement phase was more frequently observed in the histological subgroup, corresponding to a higher histological grade by definition. Also, intratumoral‐necrosis like presentation, larger peritumoral edema, and more explicit tumor margin were more frequently observed in the histological subgroup. Notably, molecular WHO Grade 4 astrocytoma showed a better prognosis, presented by a relatively long overall survival; however, no statistical significance was archieved (47 vs. 25 months, *p* = 0.22). *TP53*, *CDK6*, and *PIK3CA* alterations were commonly observed in the molecular subgroup. *PIK3R1*, *Notch1*, and *Mycn* alterations might affect the overall survival of molecular WHO Grade 4 astrocytomas. Therefore, research on potential interactions between *PIK3R1/Notch1/Mycn* alteration and *CDKN2A/B* homozygous deletion may shed light on the mechanism and therapeutic target on molecular astrocytoma, *IDH*‐mutant, Grade 4.

The WHO classification system has evolved as our understanding of gliomas has grown. Due to the close relationship between tumorigenesis and development, molecular pathogenesis has received much attention.^8^, ^9^, ^10^, ^11^, ^39^, ^40^, ^41^, ^42^, ^43^, ^44^ As *IDH1/2* mutation status predated the knowledge of astrocytic tumor prognosis, homozygous deletion of *CDKN2A/B* was further proposed to be related to a poorer prognosis in patients with astrocytoma, *IDH*‐mutant, Grade 4.^8^, ^45^, ^46^, ^47^, ^48^, ^49^ Taking *CDKN2A/B* into the evaluation scheme of *IDH*‐mutant astrocytoma specifies a more straightforward prognosis‐directed treatment pathway for various patients.^48^ However, whether the new classification effectively subgroups patients with similar prognoses remains questionable. One study from Pittsburgh found that survival was only affected by homozygous *CDKN2A* deletion in histological Grade 4 astrocytomas but not in Grades 2, 3.^50^ As suggested from our cohort analysis, patients with molecular WHO Grade 4 astrocytoma tend to have a longer survival expectancy compared to those with histological WHO Grade 4 astrocytoma. Restricted by the total number of involved cases, the difference was not statistically significant. Other confounding factors, such as follow‐up treatment and start‐up physical status, may also impact overall survival in both groups. Therefore, further investigation is required to determine the differential outcomes for histological and molecular WHO Grade 4 astrocytomas.

*PIK3R1* and *Notch1* mutations have been previously identified in patients with glioblastoma and low‐grade astrocytoma, correlating with shorter overall survival.^26^, ^51^ In addition, somatic mutations of *PIK3R1* are suggested to promote gliomagenesis.^52^ WHO Grade 4 astrocytoma patients with *PIK3R1* mutations are expected to benefit from *PI3K* inhibitors.^53^ Depending on *Mycn*, tumor‐related MiR‐29b is demonstrated to inhibit the growth of glioma.^54^ On the other hand, targeting *Mycn* can sensitize gliomas to anti‐tumor treatment.^55^ Therefore, glioma patients with *Mycn* mutations may respond to the combination of *PARP* and *CHK1* inhibitors.^56^ The finding of *PIK3R1*, *Notch1*, and *Mycn* alterations as negative prognostic factors in molecular WHO Grade 4 astrocytomas may shed light on understanding the biobehavioral progression and also potential targeted therapies for this specific subset of astrocytomas.

However, our study has certain noted limitations. First, systematic bias is inevitable, considering the small number of participants. Also, the bias induced by the small sample size affected the accuracy and reliability of the molecular subgroup analysis, therefore may influence the occurrence of possible statistical significance. Second, some histological specimens and radiological results were lost due to storage issues, further causing selective bias. Lastly, the molecular marker panel applied in this study was based on published papers containing 60 tumor‐related molecular biomarkers. Also, three patients showed enhancement in MRI underwent subtotal resection. Therefore, the possibility of undersampling may affect the accuracy of radiologic features. In follow‐up studies, more biomarkers that correlate to the prognosis of *IDH*‐mutant, Grade 4 astrocytomas, including tumor group‐specific genomic methylation profiles and copy number variations (CNV), should be considered and evaluated.

To conclude, our study scrutinized the new classification of *IDH*‐mutant, Grade 4 astrocytoma, and provided preliminary data for stratifying the effect of the WHO CNS 5 classification. Therefore, further and more precise classification should be considered, given the differential prognosis presented by histological and molecular astrocytomas. Furthermore, personalized therapies consisting of targeted molecular inhibitors for *PIK3R1*, *Notch 1*, and *Mycn* may exhibit beneficial effects in the corresponding population.

## AUTHOR CONTRIBUTIONS

**Wenlin Chen:** Formal analysis (equal); investigation (equal); writing – review and editing (supporting). **Siying Guo:** Formal analysis (equal); investigation (equal); project administration (equal); writing – original draft (lead); writing – review and editing (lead). **Yaning Wang:** Data curation (equal); formal analysis (equal); writing – review and editing (equal). **Yixin Shi:** Data curation (equal); formal analysis (equal); writing – review and editing (equal). **Xiaopeng Guo:** Investigation (equal); methodology (equal); project administration (equal). **Delin Liu:** Data curation (equal). **Yilin Li:** Data curation (equal). **Yuekun Wang:** Data curation (equal). **Hao Xing:** Data curation (equal). **Yu Xia:** Data curation (equal). **Junlin Li:** Data curation (equal). **Jiaming Wu:** Data curation (equal). **Tingyu Liang:** Data curation (equal). **Hai Wang:** Data curation (equal). **Qianshu Liu:** Data curation (equal). **Shanmu Jin:** Data curation (equal). **Tian Qu:** Data curation (equal). **Huanzhang Li:** Data curation (equal). **Tianrui Yang:** Data curation (equal). **Kun Zhang:** Data curation (equal). **Yu Wang:** Conceptualization (equal); funding acquisition (equal). **Wenbin Ma:** Conceptualization (equal); funding acquisition (equal).

## FUNDING INFORMATION

This work was supported by Beijing Municipal Natural Science Foundation (7202150) and the National High Level Hospital Clinical Research Funding (2022‐PUMCH‐A‐019) for Yu Wang, and by the National High Level Hospital Clinical Research Funding (2022‐PUMCH‐B‐113), Tsinghua University‐Peking Union Medical College Hospital Initiative Scientific Research Program (2019ZLH101) and Beijing Municipal Natural Science Foundation (19JCZDJC64200[Z]) for Wenbin Ma.

## CONFLICT OF INTEREST STATEMENT

The authors declared no conflict of interest.

## Supporting information


Figure S1.

Figure S2.

Table S1.

## Data Availability

Data sharing is not applicable to this article as no new data were created or analyzed in this study.
