# Supplementary figures and images for: Novel insight into histological and molecular astrocytoma, IDH‐mutant, Grade 4 by the updated WHO classification of central nervous system tumors
Source: Cancer Med. 2023 Sep 5;12(18):18666–78. doi: 10.1002/cam4.6476 (PMC10557904; doi:10.1002/cam4.6476)

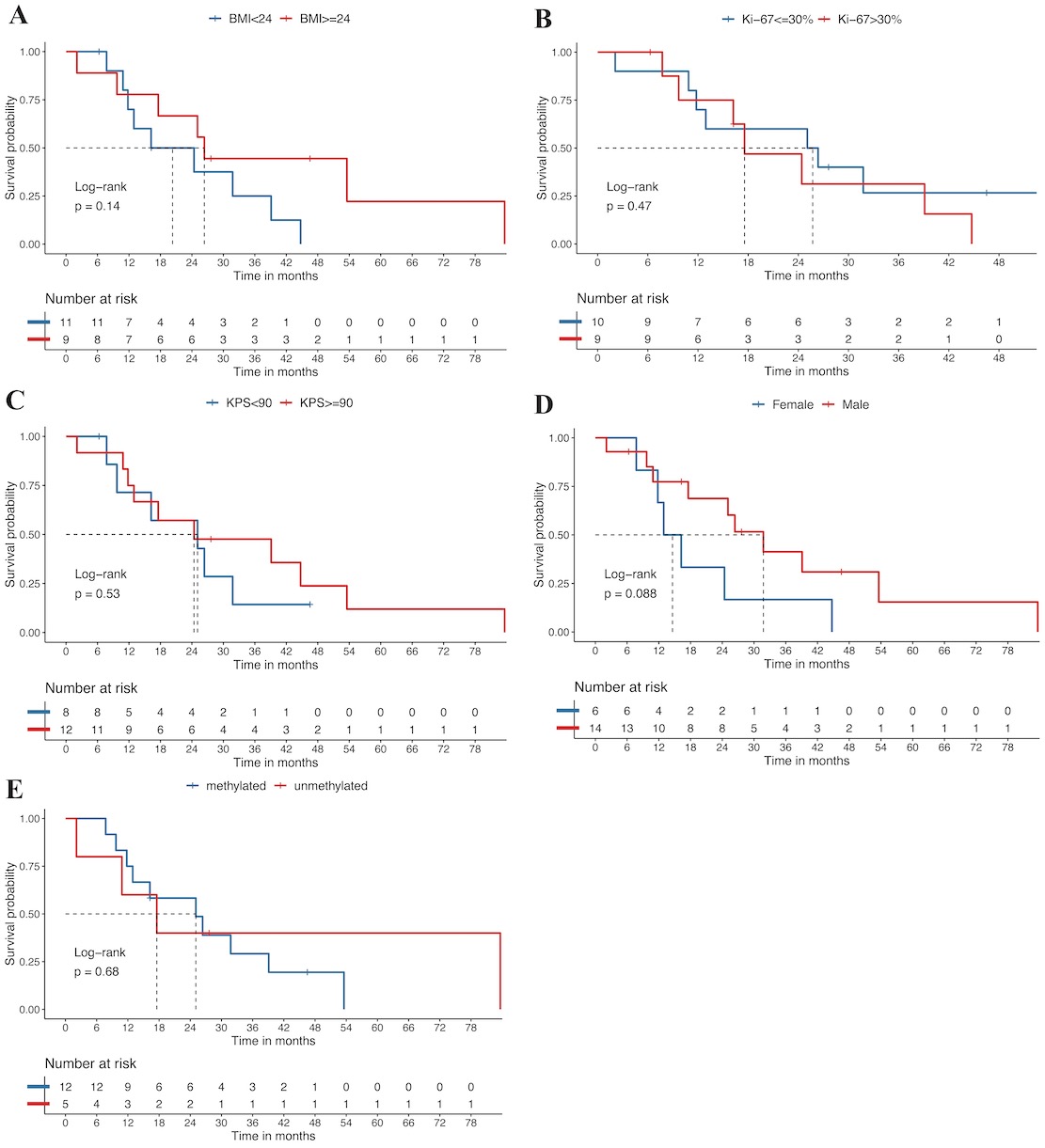

Supplement: Supplementary file 1 — Figure S1. [file CAM4-12-18666-s001.tif]

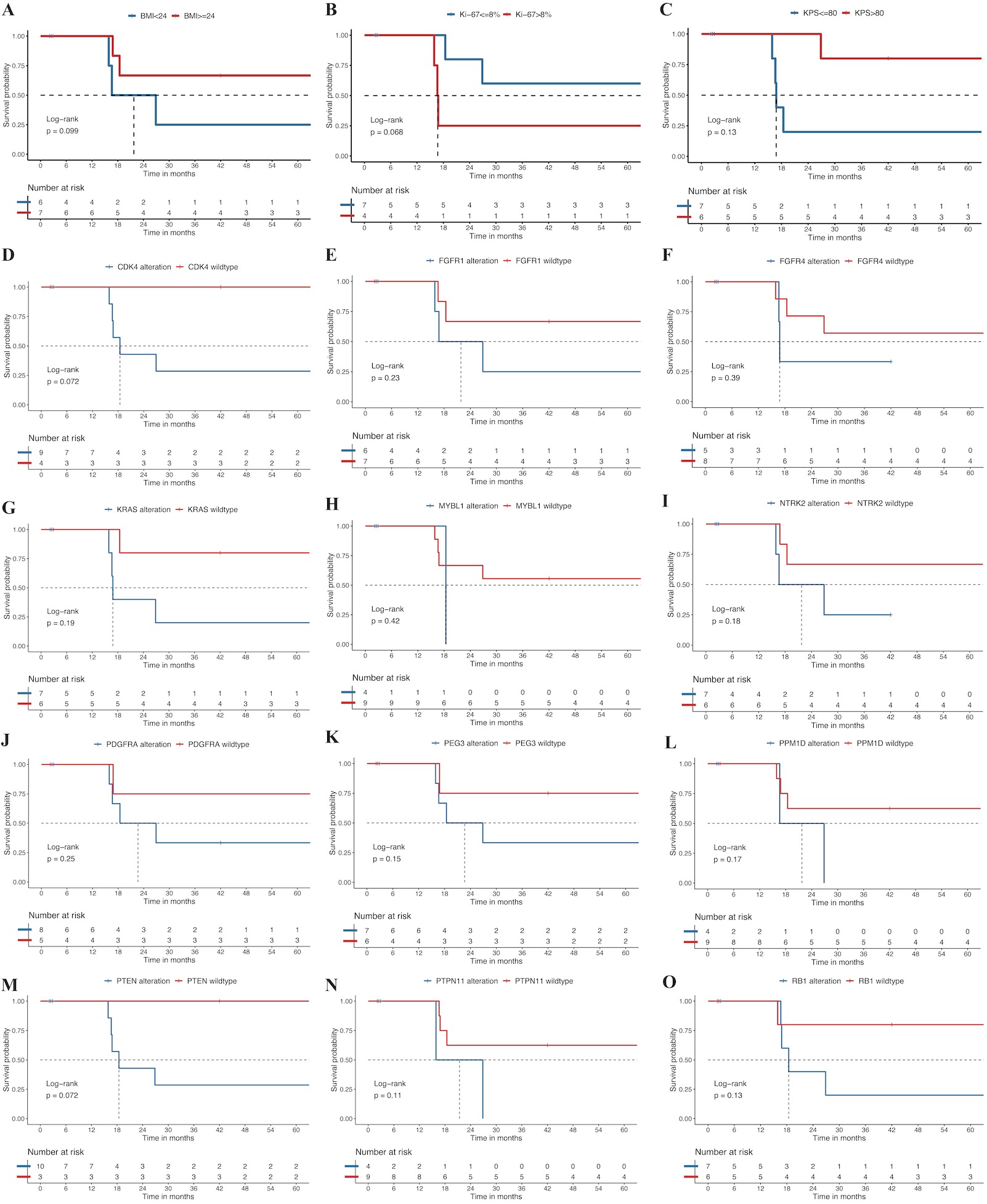

Supplement: Supplementary file 2 — Figure S2. [file CAM4-12-18666-s002.tif]
