# Supplementary material for: Novel insight into histological and molecular astrocytoma, IDH‐mutant, Grade 4 by the updated WHO classification of central nervous system tumors
Source: Cancer Med. 2023 Sep 5;12(18):18666–78. doi: 10.1002/cam4.6476 (PMC10557904; doi:10.1002/cam4.6476)
Supplement: Supplementary file 3 — Table S1. [file CAM4-12-18666-s003.docx]

Supplementary Table 1. The complete list of molecular markers.

| List of molecular markers. | | | | | | | | | |
| --- | --- | --- | --- | --- | --- | --- | --- | --- | --- |
| ACVR1 | ATRX | BCOR | BRAF | CDK4 | CDK6 | CDKN2A | CDKN2B | chr10p | chr10q |
| chr17 | chr19q | chr1p | chr7p | chr7q | chr9p | CIC | EGFR | FBXW7 | FGFR1 |
| FGFR2 | FGFR3 | FGFR4 | FUBP1 | H3F3A | HIST1H3B | HIST1H3C | IDH1 | IDH2 | KIT |
| KMT5B | KRAS | MAP2K1 | MET | MYB | MYBL1 | MYC | MYCN | NF1 | NOTCH1 |
| NRAS | NTRK2 | NTRK3 | PDGFRA | PEG3 | PIK3CA | PIK3CB | PIK3R1 | PPM1D | PTEN |
| PTPN11 | RB1 | SMARCA4 | SMARCB1 | TERT | TOP3A | TP53 | TSC1 | TSC2 | YAP1 |
